# Surgical Bailout Therapy after Implantation of a Medtronic CoreValve Bioprosthesis

**DOI:** 10.1155/2012/387103

**Published:** 2012-07-19

**Authors:** Rita Calé, José Neves, Rui Teles, João Brito, Miguel Abecasis, Manuel Almeida, Tiago Nolasco, Miguel Mendes

**Affiliations:** ^1^Cardiology Department, Santa Cruz Hospital, Lisbon, Portugal; ^2^Cardiac Surgery Department, Santa Cruz Hospital, Lisbon, Portugal

## Abstract

Moderate-to-severe paraprosthesic leak causing hemodynamic deterioration and left ventricular remodeling can occur after transcatheter aortic valve implantation (TAVI). We present the case of a 75-year-old woman who underwent TAVI with a 26 mm CoreValve prosthesis complicated with an acute left ventricle dilatation due to a severe paravalvular leak. Patient was unresponsive to elective balloon post-dilatation, and therefore she was successfully treated with open-heart surgery to remove the malfunctioning CoreValve bioprosthesis and perform standard aortic valve replacement.

## 1. Introduction

Moderate-to-severe paraprosthesic leak causing hemodynamic deterioration and left ventricular remodeling can occur after transcatheter aortic valve implantation (TAVI) [1]. There are several percutaneous techniques already described to manage early implant failure [2]. Nevertheless, the surgical bailout therapy has not been described after TAVI using the Medtronic Core Valve bioprosthesis (Medtronic, Minneapolis, MN).

## 2. Case Report

**Figure 1:**
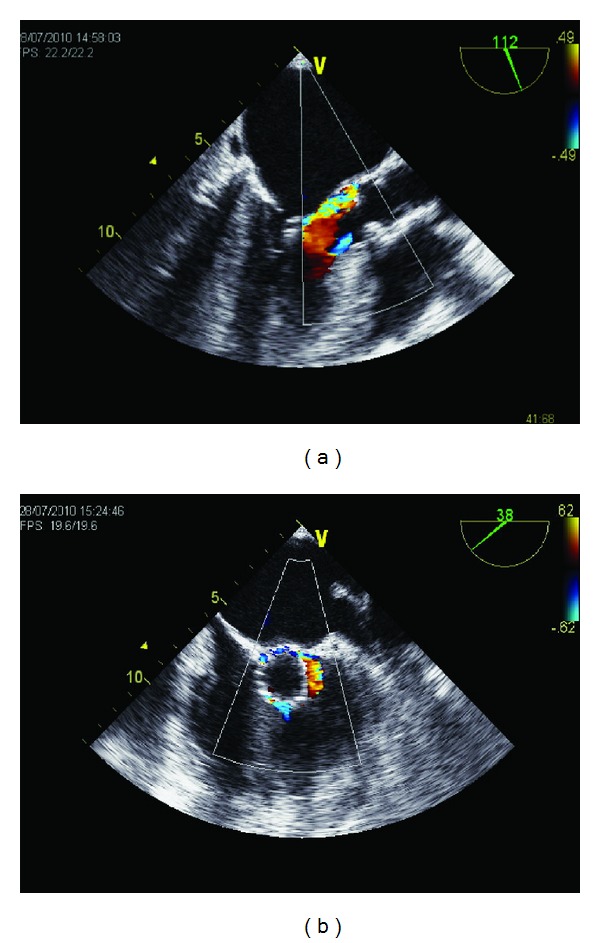
Transesophageal echocardiography images (intercomissural long axis view (a), short axis view (b)) after transcatheter valve implantation demonstrated a moderate paravalvular aortic regurgitation.

**Figure 2:**
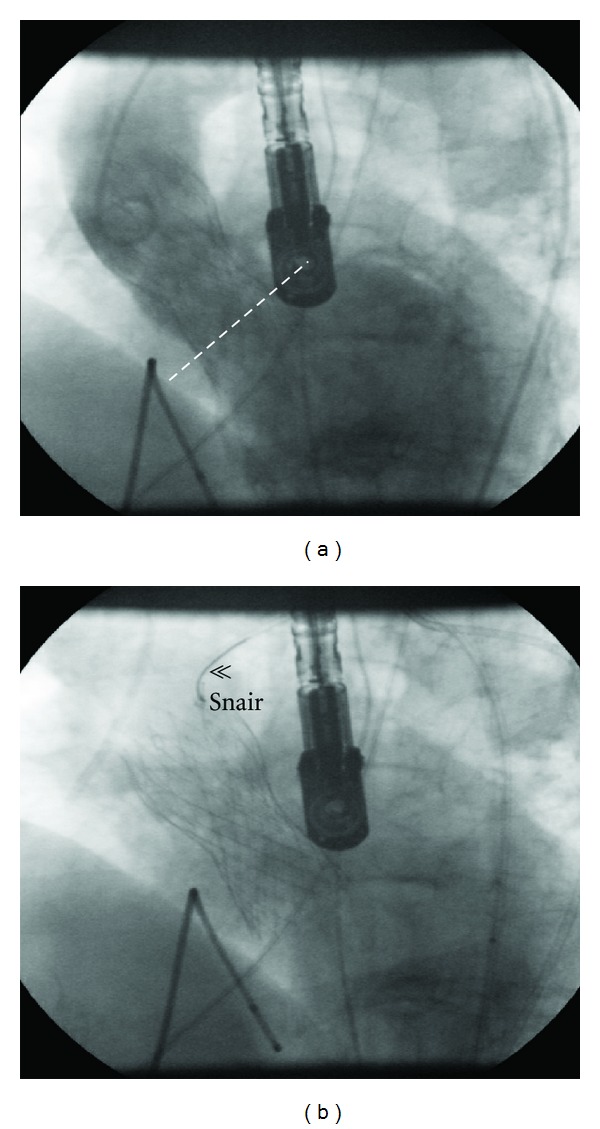
Fluoroscopy and angiographic images showing a deep implantation of the 26 mm CoreValve prosthesis (a) and its repositioning with a snair (b).

A 75-year-old female with worsening exertional dyspnea and angina for the previous 10 months was referred for cardiology evaluation. She had several cardiovascular risk factors (hypertension, diabetes, hiperlipidaemia and a morbid obesity with a BMI of 40), chronic lung disease, and renal chronic insufficiency with a calculated creatinine clearance of 38 mL/min. On the transthoracic echocardiogram, a severe stenosis aortic was diagnosed with an aortic orifice area of 0,7 cm^2^ and a systolic left ventricle dysfunction with an ejection fraction of 38%. Coronary angiography showed coronary arteries without lesions and good femoral accesses. The intraoperative transesophageal echocardiogram confirmed a heavy calcified aortic valve with an annulus of 21 mm. The transfemoral implantation of the 26 mm CoreValve prosthesis was complicated with acute moderate aortic leak (Figure 1) and moderate mitral regurgitation associated to a deep prosthesis implantation. Repositioning the prosthesis with a snair was performed to reduce the severity of mitral regurgitation with slight improvement (Figure 2). The postoperative course was uneventful, and the patient was discharged eight days later. 

**Figure 3:**
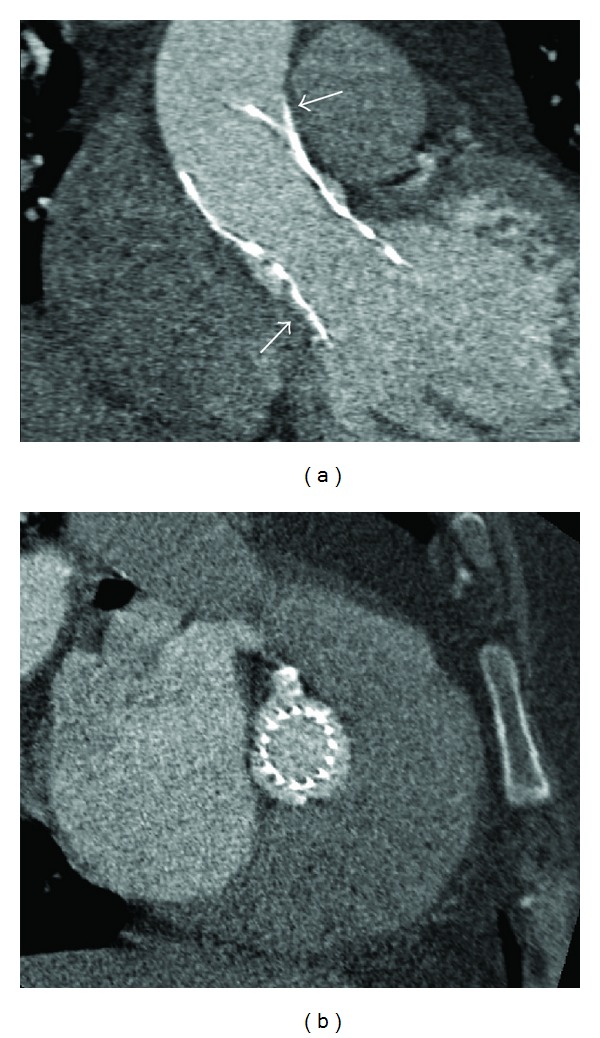
Angiotomography view showing a 26 mm CoreValve prosthesis in long-axis (a) and short-axis view (b) in an adequate position although underexpansion (white arrows).

**Figure 4:**
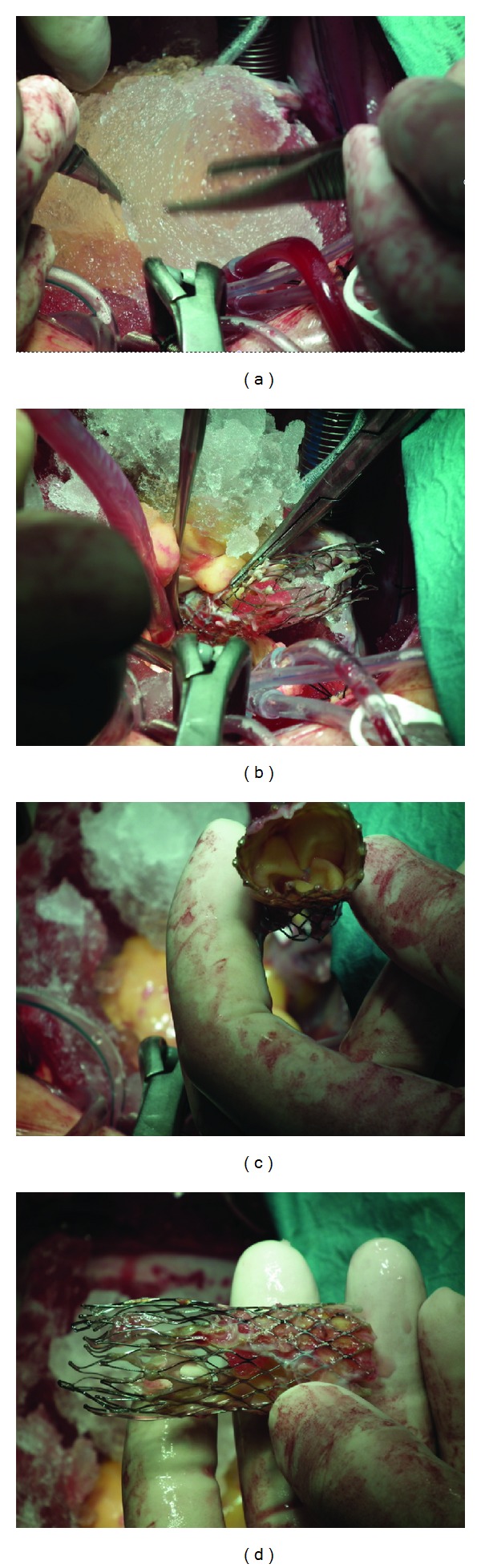
Intraoperative images. Ice was applied (a) to reduce the size of the prosthesis in order to remove it completely (b), (c ) and (d).

One month later the patient developed a progressive dyspnea and was readmitted with acute pulmonary edema. Transthoracic echocardiography evidenced a moderate-to-severe aortic paraprostesic leak (which occupies a quarter of the sewing ring, with a steep jet deceleration slope of 6,3 m/s^2^ and a holodiastolic flow reversal in the descending thoracic aorta) [3] associated with a severe left ventricle dilatation and dysfunction (left ventricle diastolic dimensions in paraesternal long axis increased from 53 to 70 mm by M-mode tracing preprocedure and postprocedure, respectively). She performed angiotomography which suggests an adequate position prosthesis although underexpansion (Figure 3). For that reason a postdilatation with a Nucleus NuMED balloon 22 mm was done without clear clinical or echocardiography improvement. The patient had a decompensated heart failure refractory to medical treatment and, despite the high-operative risk, we decided to perform an aortic valve replacement via standard median sternotomy. The 26 mm CoreValve prosthesis was removed and replacement of the aortic valve using 21 mm Mitroflow was done (Figure 4). The postoperative period was complicated by worsened renal failure requiring dialysis and paroxysmal atrial fibrillation but the patient was discharged without dyspnea two months later. Transthoracic echocardiography at discharged showed a normal prosthetic valve function and a slight improvement on left ventricle dimensions and function, although not completely normal.

## 3. Discussion

After TAVI, a significant paravalvular leak can occur for several reasons: undersized prosthesis, malposition of the device (very deep or high implantation), prosthesis under-expansion, the presence of heavily calcified stenotic aortic valve or a bicuspid valve [4]. With the currently available devices, it is very difficult to fully reposition the bioprosthesis after initial deployment. Several bailout transcatheter techniques have been described to manage suboptimal prosthesis deployment: a postdilatation, snair repositioning, or valve-in-valve technique with implantation of a second device inside the first one [2, 4–7]. In the present case, there was more than one possible mechanism for the development of this severe paravalvular leak: the first explanation was deep valve implantation, but this was resolved intraprocedure by repositioning the prosthesis with a snair and documented by angio-CT. A second possible mechanism was underexpansion, as angiotomography scan evidenced, but a postdilatation did not improve clinical or echocardiography status. This was probably related to insufficient oversizing of the transcatheter valve with respect to the patient's aortic annulus and the severity and asymmetric distribution of valve calcification. The implantation of a second valve might be associated with an increase of the radial force [6], although “valve-in-valve” procedure, eventually with a balloon expandable, is mainly indicated to acutely manage device malposition.

Despite the absence of such case reports, our heart team decided to undergo an open-heart surgery, to remove the malfunctioning CoreValve bioprosthesis and perform standard aortic valve replacement. Cardiac surgery was successful, although complicated by acute renal failure requiring dialysis. We can also discuss percutaneous valve-in-valve procedure as an alternative strategy. 

Presently there is scarce data on paravalvular leak predictors. As other authors have already suggested [4, 6], there is a need to better predict and assess ominous prognosis caused by paravalvular leaks after TAVI and to determine the role of different strategies for the treatment of each mechanism.

## 4. Conclusion

The surgical bailout therapy by implanting a second aortic bioprosthesis through conventional surgery after removing the first malfunctioning CoreValve bioprosthesis in a high-risk patient is possible, with good results and without major complications. In the future, the development of percutaneous bioprosthesis that reduce paravalvular leaks and allow valve retrieval and reposition after suboptimal deployment could avoid the need of surgical bailout therapy.
